# Can the Profitability of Medical Enterprises Be Improved After Joining China's Centralized Drug Procurement? A Difference-in-Difference Design

**DOI:** 10.3389/fpubh.2021.809453

**Published:** 2022-02-01

**Authors:** Yu-Fei Hua, Jin Lu, Bing Bai, Han-Qing Zhao

**Affiliations:** ^1^School of Economics, Qingdao University, Qingdao, China; ^2^Shandong Vocational College of Information Technology, Weifang, China; ^3^ABB China, Beijing, China

**Keywords:** China's centralized drug procurement, enterprise profits, generic drug, original drug, difference-in-difference

## Abstract

This paper explores the impact of joining centralized drug procurement of China on the profitability of medical enterprises by the difference-in-difference (DID) model. When centralized procurement cannot bring enough cost savings to enterprises, the price competition caused by centralized procurement will lead to the decline of enterprise profits. In the short term, the negative impact of China's drug centralized procurement policy on the net profit of enterprises is not obvious in the year when enterprises win the bid. After the government officially purchases from pharmaceutical enterprises, the negative impact of the drug centralized procurement policy of China on the net profit of enterprises begins to appear gradually. Therefore, the generic drug manufacturers increase R&D investment and have their own heavy products of original drugs as soon as possible to enhance their core competitiveness.

## Introduction

On May 11, 2021, China's National Bureau of Statistics released the data of the seventh national census. In terms of population composition, the population aged 60 and over was 264.02 million that accounted for 18.70%, an increase of 5.44% compared with 2010. With the aggravation of aging, the number of patients with chronic diseases is increasing, and the high drug price leads to the increasing medical burden of patients and the country. Medical insurance cost control is a very important means to reduce the overall burden by controlling drug prices. China's generic drugs and patent expired original drugs account for more than half of the market share in terms of sales quantity and amount. In order to promote market competition, drive the upgrading of the pharmaceutical industry and effectively reduce the drug burden of patients, the state launched the pilot of “4 + 7” urban drug centralized procurement in December 2018. For “4 + 7” centralized procurement, the State shall agree on the sales volume of drugs in advance, adopt the form of exclusive bid winning, ensure the priority use of bid-winning drugs, and encourage enterprises to reduce prices and bid. Most of the winning products are also products in the field of chronic diseases. In the past, most of the expenditure on medical insurance was spent on drugs, and a considerable part was given to auxiliary drugs, expired patented drugs, and domestic generic drugs. If this area is not adjusted, innovative drugs and imported drugs will not enter medical insurance, which is unreasonable to be solved urgently. Now, auxiliary drugs have been strengthening supervision, and the overall direction is to adjust the drug structure of medical insurance. Therefore, it is also a natural thing to purchase with volume before the reform and under the general trend.

For the pharmaceutical industry of China, the era of high gross profit of generic drugs is over, and a reasonable profit margin of the manufacturing industry will be earned in the future. Medicare will make more space for covering patented innovative drugs. The national centralized procurement of drugs with volume brings a one-time market increment opportunity for most varieties with low market share. The varieties listed can directly obtain more than 60% of the market in 11 pilot cities in centralized procurement, and the market pattern of some varieties will change dramatically. However, because the pharmaceutical market is not a fully market-oriented market, supply exceeds demand, the buyer is strong, and the buyer is limited by policies and itself. In addition, there are more influencing factors in the prescription process. The impact on the profitability of pharmaceutical enterprises after joining China's centralized drug procurement needs to be observed and digested by the market. The impact of this reform on different enterprises is different. There are several situations for the bid-winning enterprise. If the market share of the bid-winning products is not too high before entering the catalog, 60–70% of the sales volume of the bid-winning market can be changed. Changing price for quantity can quickly increase the sales volume of products and occupy most of the market share. If the market share is high before, and the price reduction is large during the negotiation process, the amount exchanged at the price may not be enough to make up for the loss of the price. For example, the bid price of liver disease drug Runzhong produced by Zhengda Tianqing has been reduced by more than 90%, which may affect the product profit in the short term. For exclusive varieties, due to the small number of bargaining enterprises, the reduction space may not be too large, and there are great opportunities for improvement in the later stage. After pharmaceutical enterprises join the national centralized procurement, can the profitability of pharmaceutical enterprises be improved by exchanging quantity for price? This is the problem to be discussed in this paper.

The study makes two contributions. First, based on the pharmaceutical market of China, this paper makes an in-depth study on the decision-making behavior of pharmaceutical enterprises and centralized procurement platforms. Explore the impact of enterprise bargaining power and cost advantage of centralized procurement on the willingness of pharmaceutical enterprises to participate in centralized procurement. Analyze the impact mechanism of centralized procurement regulation on drug purchase price and drug market performance. Based on this, it puts forward the conditions for double oligarch competitive pharmaceutical enterprises to achieve a “win-win” under the centralized procurement regulation. Secondly, centralized drug procurement policy of China is a comprehensive pilot policy popularized on a small scale throughout the country. However, up to now, the evaluation of the effect of this policy has almost stayed in qualitative analysis. If we can make a profound and effective quantitative evaluation of the policy effect, it will be of great significance in both theory and practice. Therefore, this paper intends to carry out exploratory research.

The rest of this study is organized as follows: “Literature Review” reviews the existing literature. Whereas Section “Nash Equilibrium Model of Duopoly Competition” presents the influence mechanism of centralized procurement regulation on drug purchase price and drug market performance. Section “Methodology and Data” describes the difference-in-difference (DID) model in this paper and the data description. Section “Empirical Results” shows the findings of the study. Section “Conclusions” offers concluding remarks.

## Literature Review

Centralized drug purchase is the key link to reduce drug prices, rectify drug circulation orders, and standardize drug use. The American Medical Supply Chain Association reported that centralized procurement can save 10–35% of procurement expenditure for medical institutions every year (1). Unlike the centralized drug procurement in the United States, which is completely configured by the market, centralized drug procurement of China is a supporting policy in the national medical system reform. It aims to regulate the purchase and sale of drugs through administrative means, which has obvious characteristics of government regulation (2). Centralized procurement organizations negotiate with upstream pharmaceutical enterprises through the centralized formation of large-scale orders to obtain high discounts to reduce drug purchase prices (3). In centralized procurement negotiations, group purchasing organization (GPO) has complete bargaining power, but this is not consistent with the reality (4). In fact, the formation of drug prices in China is closely related to the bargaining power of pharmaceutical enterprises and the willingness of platform participation (5). Zhang et al. (6) studied the pricing and channel performance of platform enterprises under group purchase strategy under the condition of competition, which provides a better model and method for this study. The bidding mechanism of centralized procurement can be summarized as the process of the government agreeing on the procurement volume and identifying the enterprises with the lowest cost. In the case of asymmetric information, such “quantitative auction” is a convenient but suboptimal procurement strategy (7). There is an optimal procurement strategy based on the quantitative auction.

In the early research on centralized procurement in China, many scholars discussed the impact of centralized procurement policy from the perspectives of theoretical analysis, the price mechanism, and policy interpretation. Li et al. (8) studied the root causes of falsely high drug prices caused by the original drug bidding mechanism from the perspective of the theoretical model and gave suggestions on the mechanism design in the future. Chen et al. (9) expounded the economic theoretical basis of “4 + 7” centralized procurement from the perspective of a single source of goods, buyer monopoly, and seller monopoly, analyzed the implementation effect of the policy, and warned that we should carefully avoid the possibility of “bad money expelling good money”. Wang et al. (10) established a game model to predict the optimal bidding price of enterprises based on the results of “4 + 7” centralized procurement and the assumption of triangular probability density. Yang et al. (11) started with the quality cost indifference curve and proposed that “4 + 7” centralized procurement is a Pareto improvement of general centralized procurement. Taking hypolipidemic drugs as an example, Hu et al. (12) analyzed the changing characteristics of drug market of China under the new policy of medical reform, such as centralized procurement, and predicted the future market. Based on the Raci analysis model, Chen et al. (13) substituted the relevant parties of centralized procurement into different roles, discussed the performance, supervision, and cooperation mechanism of centralized procurement and found the difficulties and key points in the implementation of the policy.

There are three main types of related research. The first category is the research on centralized drug procurement. Among them, part of the literature expounds the function and principle of the centralized procurement organization to reduce drug prices (14–17). As an intermediary organization between the supplier and the demander, the centralized procurement organization reduces the drug purchase price by reducing transaction costs and promoting competition among pharmaceutical enterprises. The other part of the literature mostly analyzes the existing problems and puts forward policy suggestions for domestic scholars (18–21). The second category is the research on the effect of government behavior on enterprise net profit. Among them, there are three different views: one is that government centralized purchase promotes the growth of enterprise net profit through internal incentives (22, 23). The other believes that due to information asymmetry, government intervention has an inhibitory effect on enterprise net profit (24, 25). In addition, some scholars believe that the impact of centralized government drug procurement on the net profit of enterprises is uncertain. The centralized procurement of generic drugs will make enterprises increase innovation investment in the short term to reduce the net profit. However, in the long run, the original drug developed by the enterprise can improve the net profit of the enterprise (26, 27). The third category is the research on the voluntary supply of public goods. The social welfare brought by encouraging enterprise R& D and innovation can be regarded as a kind of public goods provided by the government to consumers, with non-exclusive characteristics. No matter whether an individual government supplies or not and how much it supplies, it can benefit from the improvement of drug quality. This leads to the problem of insufficient voluntary supply of public goods. The problem in the global drug market is that governments try to bear only the marginal cost of domestic drug consumption and rely on other countries to pay for the globally shared innovative achievements in drug R&D. From the perspective of cooperative organizations, with the reduction of the number of individual members, it is less difficult to punish the “free rider” behavior in the organization, and the uncooperative behavior is easier to be detected. Therefore, the level of voluntary supply of public goods is higher (28, 29).

Previous literature provides some enlightenment. First of all, although net profit is an element of the development of pharmaceutical enterprises. However, it is worth discussing whether it can benefit from the government's centralized drug procurement policy to achieve the growth of enterprise net profit. Secondly, the existing research has not discussed the bargaining game model of bargaining power and procurement cost. It also does not involve the impact of pharmaceutical market regulation factors on drug prices and market performance. Based on the pharmaceutical market of China, this paper makes an in-depth study on the decision-making behavior of pharmaceutical enterprises and centralized procurement platforms. Explore the impact of enterprise bargaining power and cost advantage of centralized procurement on the willingness of pharmaceutical enterprises to participate in centralized procurement. Analyze the impact mechanism of centralized procurement regulation on drug purchase price and drug market performance. Based on this, it puts forward the conditions for double oligarch competitive pharmaceutical enterprises to achieve a “win-win” under the centralized procurement regulation. Finally, the existing research only focuses on the decline of the net profit of pharmaceutical enterprises. However, for a long time, the evaluation of profitability has only paid attention to financial indicators and only used the relevant data of financial reports to evaluate the profitability of enterprises. It ignores the evaluation of the impact of centralized drug procurement policy of China on the stability and sustainability of enterprise profitability. Therefore, it is necessary to consider the policy effect of the net profit growth of pharmaceutical enterprises.

## Nash Equilibrium Model of Duopoly Competition

Considering the duopoly competition in the pharmaceutical market, medical enterprises (recorded as A and B) produce and sell innovative drugs with certain substitutes to downstream medical institutions, such as drugs of different brands for the treatment of the same kind of diseases. In order to study the profitability of medical enterprises after joining centralized drug procurement of China, this paper considers non-centralized procurement and centralized procurement and takes the former as the benchmark (30).

### Construction of Market Demand Model

Consider the Linear Hotelling model to describe the heterogeneous purchase preference of medical institutions. The preferences of medical institutions depend on their location on the Hotelling line. Therefore, the medical institution located in *X* ∈ [0, 1] purchases drugs from A and B, and the utility obtained is

(1) $$\begin{array}{r} U_{A}=V_{A}-tx-p_{A}-c_{p}^{A}\\ U_{B}=V_{B}-t\left(1-x\right)-p_{B}-c_{p}^{B} \end{array}$$

Where *V*_*i*_ is the maximum willingness of medical institutions to pay for drugs *i, t* is unit distance cost, *p*_*i*_ is the purchase price of drugs *i*, $c_{p}^{i}$ is the unit transaction cost of medical institutions under different procurement modes.

Given *p*_*i*_ and transaction mode, the market demands of enterprises A and B are:

(2) $$\begin{array}{r} q_{A}=x=\frac{1}{2}+\frac{p_{B}-p_{A}+c_{p}^{B}-c_{p}^{A}}{2t}\\ q_{B}=1-x=\frac{1}{2}+\frac{p_{A}-p_{B}+c_{p}^{A}-c_{p}^{B}}{2t} \end{array}$$

### Bargaining Game Between Enterprises and Centralized Procurement Platform

This paper constructs a two-person bilateral bargaining game model between duopoly competitive enterprises and procurement platforms. The model consists of two bargaining units:


(3) $$\begin{array}{r} \left\{\left(enterprise1,platform\right),\left(enterprise2,platform\right)\right\} \end{array}$$

The bargaining result of each unit can be determined by the asymmetric Nash bargaining solution. Since the two enterprises negotiate with the same platform, the values of *D*_*i*_ and *d*_*i*_ depend on the negotiation results between competitors and the platform. Where *D*_*i*_ and *d*_*i*_ are the breakdown points of negotiation between enterprise *i* and platform, respectively, that is, the profits of both parties when the agreement is not reached. Therefore, the feasible set of enterprise *i* and platform bargaining solution is:

(4) $$\begin{array}{r} \Xi_{i}\left(p_{i},p_{j},\prod_{j}\right)=\{\left(\prod_{i},\pi \right):\prod_{i}\geq D_{i},\pi \geq d_{i},\prod_{i}\\ +\pi \leq G_{i}+G_{j}-\prod_{j}\} \end{array}$$

∏_*i*_, ∏_*j*_, and π are respectively used to express net profit of enterprises *i*, enterprises *j*, and platforms., and *G*_*j*_ are respectively the total profits of the enterprise and the platform when enterprise *i* and enterprise *j* reach a centralized purchase agreement with the platform. The Nash bargaining solution between enterprise *i* and the platform is:

(5) $$\begin{array}{r} \underset{\prod_{i},\pi \in \Xi_{i}}{\max}\left(\prod_{i}-D_{i}\right)\alpha_{i}+\left(\pi -d_{i}\right)\left(1-\alpha_{i}\right)\\ s.t.\prod_{i}\geq D_{i},\pi \geq d_{i} \end{array}$$

### Model Solving and Analysis

There are four possible bargaining results for enterprises and platforms: (no participation, no participation), that is, neither enterprise participates in centralized procurement, [participation (non-participation), non-participation (participation)], that is, one enterprise participates in centralized procurement and the other does not participate, and (participation, participation), that is, both enterprises participate in centralized procurement. DD, CD, DC, and CC are respectively used to represent the above four possible bargaining equilibria.

Given the decision of competitors, the decision problem of enterprise *i* is:

(6) $$\begin{array}{r} \underset{{\text{p}}_{\text{i}}}{\text{max }}G_{i}\left(p_{i}|p_{j},{\bar{c}}_{i},{\bar{c}}_{j}\right)=\left(p_{i}-{\bar{c}}_{i}\right)\left(\frac{1}{2}+\frac{p_{j}-p_{i}+c_{p}^{j}-c_{p}^{i}}{2t}\right) \end{array}$$

In product competition equilibrium, the enterprise's pricing strategy and market demand are respectively:


(7) $$\begin{array}{l} \{\begin{array}{cc} p_{i}^{*}\left({\bar{c}}_{i},{\bar{c}}_{j},c_{p}^{i},c_{p}^{j}\right)=V-c_{p}^{i},q_{i}^{*}\left({\bar{c}}_{i},{\bar{c}}_{j},c_{p}^{i},c_{p}^{j}\right)=0\; & {\bar{c}}_{i}+c_{p}^{i}-{\bar{c}}_{j}-c_{p}^{j}\geq 3t\\ p_{i}^{*}\left({\bar{c}}_{i},{\bar{c}}_{j},c_{p}^{i},c_{p}^{j}\right)=t+\frac{2{\bar{c}}_{i}+{\bar{c}}_{j}+c_{p}^{j}-c_{p}^{i}}{3},q_{i}^{*}\left({\bar{c}}_{i},{\bar{c}}_{j},c_{p}^{i},c_{p}^{j}\right)=\frac{3t+{\bar{c}}_{j}-{\bar{c}}_{i}+c_{p}^{j}-c_{p}^{i}}{6t}\; & |{\bar{c}}_{i}+c_{p}^{i}-{\bar{c}}_{j}-c_{p}^{j}|<3t\\ p_{i}^{*}\left({\bar{c}}_{i},{\bar{c}}_{j},c_{p}^{i},c_{p}^{j}\right)=V-t-c_{p}^{i},q_{i}^{*}\left({\bar{c}}_{i},{\bar{c}}_{j},c_{p}^{i},c_{p}^{j}\right)=1\; & {\bar{c}}_{i}+c_{p}^{i}-{\bar{c}}_{j}-c_{p}^{j}\leq -3t \end{array} \end{array}$$

Where *i* = *A, B, j* ≠ *i*.

When enterprise B fails to reach an agreement with the platform, the Nash bargaining solution between enterprise A and the platform is:

(8) $$\begin{array}{r} \{\begin{array}{c} \prod_{A}^{CD}=\frac{\left(1-\alpha_{A}\right){\left(3t+\Delta_{B}-\Delta_{A}\right)}^{2}+\alpha_{A}\left(3t+c_{e}+\Delta_{B}\right)\left(3t-2c_{e}+\Delta_{B}\right)}{18t}\\ \pi^{CD}=\frac{\left(1-\alpha_{A}\right)\left(6t+c_{e}+2\Delta_{B}-\Delta_{A}\right)\left(c_{e}+\Delta_{A}\right)+3\alpha_{A}c_{e}\left(3t+c_{e}+\Delta_{B}\right)}{18t} \end{array} \end{array}$$

Similarly, when enterprise a fails to reach an agreement with the platform, the Nash bargaining solution between enterprise B and the platform is:

(9) $$\begin{array}{r} \{\begin{array}{c} \prod_{B}^{DC}=\frac{\left(1-\alpha_{B}\right){\left(3t+\Delta_{A}-\Delta_{B}\right)}^{2}+\alpha_{B}\left(3t+c_{e}+\Delta_{A}\right)\left(3t-2c_{e}+\Delta_{B}\right)}{18t}\\ \pi^{CD}=\frac{\left(1-\alpha_{B}\right)\left(6t+c_{e}+2\Delta_{A}-\Delta_{B}\right)\left(c_{e}+\Delta_{B}\right)+3\alpha_{B}c_{e}\left(3t+c_{e}+\Delta_{B}\right)}{18t} \end{array} \end{array}$$

When both enterprises participate in centralized procurement, the Nash bargaining between enterprise I and the platform is:

(10) $$\begin{array}{l} \{\begin{array}{c} \prod_{\text{i}}^{CC}=\frac{{\left(3t-c_{e}-\Delta_{\text{i}}\right)}^{2}}{18t}+\frac{\alpha_{j}\left[\left(1-\alpha_{i}\right)\left(6t+c_{e}+2\Delta_{j}-\Delta_{i}\right)\left(c_{e}+\Delta_{i}\right)+2\alpha_{i}\left(c_{e}+\Delta_{i}\right)\left(3t+c_{e}+\Delta_{j}\right)\right]}{18\left(1-\alpha_{i}\alpha_{j}\right)t}\\ +\frac{\alpha_{i}\left(1-\alpha_{j}\right)}{18\left(1-\alpha_{i}\alpha_{j}\right)t}\left[\left(6t+\Delta_{i}-\Delta_{j}\right)\left(3c_{e}+2\Delta_{i}+\Delta_{j}\right)-\left(3c_{e}+2\Delta_{i}\right)\left(c_{e}+\Delta_{i}\right)\right]\\ \pi^{CC}=\frac{\left(1-\alpha_{A}\right)\left(1-\alpha_{B}\right)}{18\left(1-\alpha_{A}\alpha_{B}\right)t}\left[\left(6t-c_{e}-\Delta_{A}\right)\left(c_{e}+\Delta_{A}\right)+\left(6t-c_{e}-\Delta_{B}\right)\left(c_{e}+\Delta_{B}\right)\right]+\frac{3\alpha_{A}\alpha_{B}c_{e}}{18\left(1-\alpha_{A}\alpha_{B}\right)}[\left(1-\alpha_{B}\right)\left(3t+c_{e}+\Delta_{A}\right)\\ +\left(1-\alpha_{A}\right)\left(3t+c_{e}+\Delta_{B}\right)]+\frac{\alpha_{A}{\left(1-\alpha_{B}\right)}^{2}\left(6t+c_{e}+2\Delta_{A}-\Delta_{B}\right)\left({\text{c}}_{e}+\Delta_{B}\right)+\alpha_{B}{\left(1-\alpha_{A}\right)}^{2}\left(6t+c_{e}+2\Delta_{B}-\Delta_{A}\right)\left({\text{c}}_{e}+\Delta_{A}\right)}{18\left(1-\alpha_{A}\alpha_{B}\right)t} \end{array} \end{array}$$

Finally, the optimal centralized procurement participation decision of enterprises is determined, that is, the subgame refined Nash equilibrium (SPNE) of the whole game.

When the centralized procurement efficiency of two enterprises is equal (i.e., Δ_*A*_ = Δ_*B*_), participating in centralized procurement is the optimal strategy for both enterprises. However, when the centralized procurement efficiency of the two enterprises is quite different (i.e., Δ_*A*_ ≠ Δ_*B*_), only enterprises with high centralized procurement efficiency (i.e., Δ_*A*_ > Δ_*B*_) select centralized purchase. In particular, if and only if the centralized procurement efficiency of both enterprises is high (i.e., $\Delta_{A}>{\bar{\Delta }}_{A}$ and $\Delta_{B}>{\bar{\Delta }}_{B}$), both enterprises choose to participate in centralized procurement.

Due to the imbalance in scale, market position, and cost management of medical enterprises in China, there are great differences in centralized procurement efficiency of medical enterprises (i.e., Δ_*A*_ ≠ Δ_*B*_). When centralized procurement cannot bring enough cost savings to enterprises, the price competition caused by centralized procurement will lead to the decline of enterprise profits. This well explains the reason why medical enterprises are opposed to centralized procurement regulation. Medical enterprises claim that centralized procurement regulation has squeezed their profits.

## Methodology and Data

### Methodology

In order to test the profitability of medical enterprises after joining centralized drug procurement of China, we used a DID method proposed by Maria et al. (31). According to the list published in the results of centralized drug procurement in 4 + 7 cities, 15 enterprises with 26 drugs are involved. This provides a good “quasi-natural experiment” for DID method.

The research sample selected in this paper is 65 generic drug listed companies in China, of which 6 bid-winning enterprises constitute the “experimental group”, and the other 59 non-bid-winning enterprises naturally constitute the “control group”. When using the DID method, the experimental group virtual variable *treated* is generally set according to whether it is affected by the policy. The group affected by the policy is regarded as the experimental group, which is assigned as 1 and the control group as 0. At the same time, according to the time of policy implementation, set the virtual variable *period* of experimental stages successively, assign 1 to the *period* in the year and after policy implementation, and assign 0 to the *period* before policy implementation. Accordingly, the samples can be divided into four groups: control group before policy implementation (*treated* = 0, *period* = 0), control group after policy implementation (*treated* = 0, *period* = 1), experimental group before policy implementation (*treated* = 1, *period* = 0), and experimental group after policy implementation (*treated* = 1, *period* = 1). Among them, the interaction terms of experimental grouping and experimental staging are *treated* × *period* is the net effect of policy implementation. This paper uses the dummy variable to construct a two-way fixed effect model to test the profitability of medical enterprises after joining centralized drug procurement of China (32). The specific model settings are as follows:

(11) $$\begin{array}{r} pro_{it}=\alpha_{0}+\alpha_{1}DID_{it}+\gamma x_{it}+\eta_{t}+\mu_{i}+\varepsilon_{it} \end{array}$$

*P* is the incidence of poverty, *H* is the health human capital, *control* is the control variables, and Δ is the first-order difference item;α_1_, α_2_, α_*ki*_ is the short-term dynamic relationship; α_6_, α_7_, α_*m*_ is the co-integration relationship between variables.

*pro*_*it*_ is the explanatory variable, which indicates the profit level of the *i*th enterprise in the *t* year. This paper uses the enterprise net profit to measure. DID_*it*_ is the dummy variable of drug centralized purchase policy. x_*it*_ is a group of control variables, i.e., R & D capability, growth capability, operation capability, cost management, cash flow management (CFM), marketing capability, ownership structure, and capital structure. η_*t*_ is a time fixed effect, μ_*i*_ is the individual fixed effect of each enterprise. α_1_ is the core estimation parameter, which represents the net effect on the medical enterprise's profit after joining centralized drug procurement of China. If α_1_ positive indicates that centralized drug procurement of China is indeed conducive to increasing the net profit of medical enterprises, on the contrary, it has an inhibitory effect.

### Data

This paper is based on the data of 65 listed companies in the generic drug consistency evaluation section of China's stock market for 2011–2020, and it is retrieved from China's Wind database. The choice of starting period is limited by data availability. The explanatory variable of this paper is the profitability of medical enterprises, which is measured by the net profit of enterprises. The core explanatory variable of this paper is the dummy variable *DID* (centralized drug procurement). According to the list of purchased varieties in the document of centralized drug procurement in 4 + 7 cities in 2018, combined with the unified assignment of the winning enterprises, the core explanatory variable *DID* is finally obtained. Based on the existing literature, the control variables affecting the profitability of Chinese medical companies mainly include R&D capability, growth capability, operation capability, cost management, CFM, marketing capability, ownership structure, and capital structure. The indicators of R&D capability (*RD*) are as follows: the proportion of intangible assets and the proportion of enterprise R&D personnel in the total number of employees. This paper uses the annual growth rate of total assets to measure the growth ability of enterprises (GRO). Through the accounts receivable turnover (ART) index to measure the efficiency of enterprises in using funds. The cost management (CM) indicators are measured by the ratio of operating expenses to operating revenue, the ratio of administrative expenses to operating revenue, and the ratio of financial expenses to operating revenue. The ratio of net cash flow from operating activities to operating income is used to measure the enterprise's CFM ability. The marketing ability is measured by the net profit margin on sales (SM). The ownership structure is measured by the holding proportion of the top ten shareholders (PSH). The capital structure is measured by the asset-liability ratio (*ALR*) (33–35).

**Figure 1:**
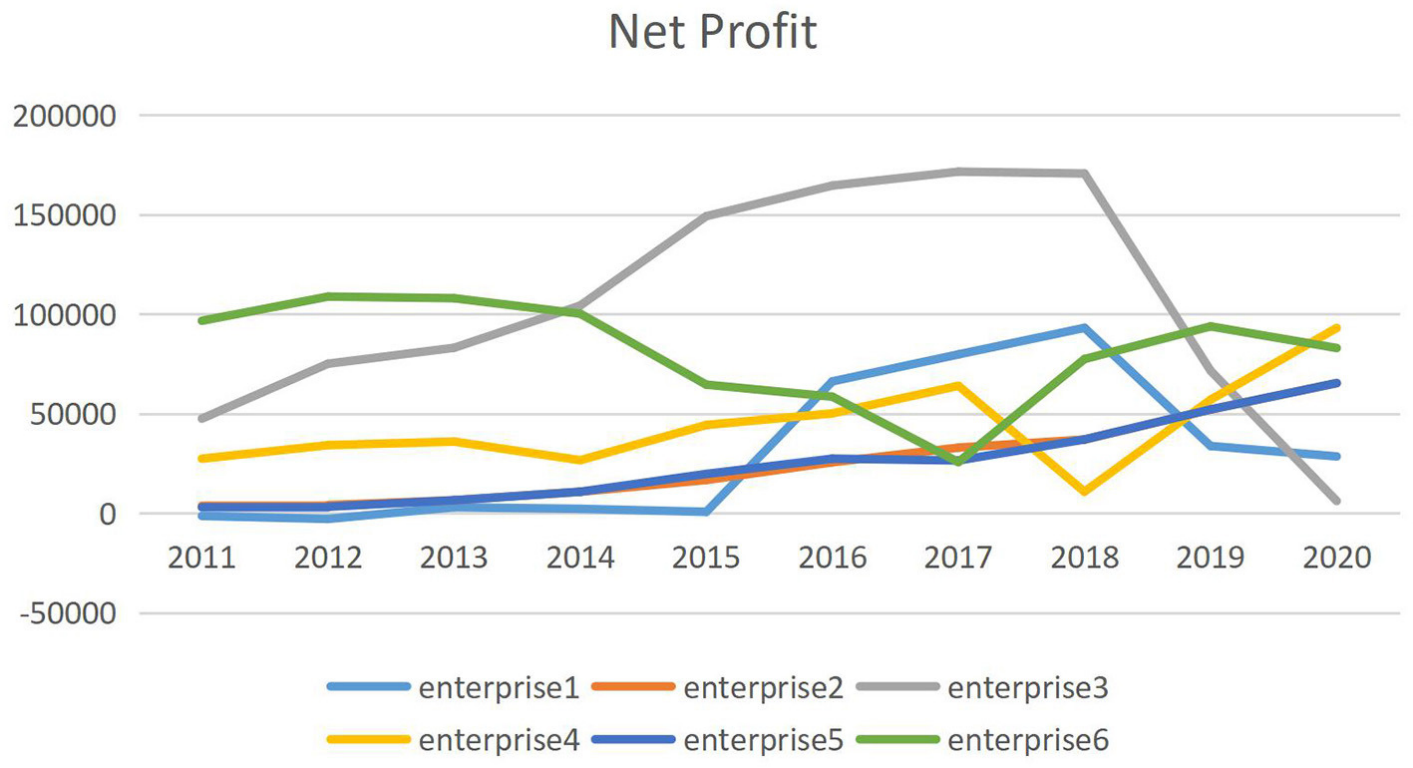
Trends in net profit of bid-winning enterprise from 2011 to 2020.

Figure 1 shows trends in the profitability of 6 listed medical enterprises that have joined centralized drug procurement of China from 2011 to 2020. It can be seen that the net profit growth trend of the six enterprises before 2018 is similar, and the performance has differentiated since the centralized purchase of drugs in 2018. The net profit of some companies began to decline, and the net profit of some companies still maintained rapid growth. Among the six enterprises, the enterprise with the highest net profit growth was increased by 764.86%, and the enterprise with the lowest net profit growth was decreased by 96.43%. Therefore, it is very important to study the reasons for the changes in the profitability of Chinese pharmaceutical companies after 2018.

In addition, we have control of variables. The medical industry is a high-tech industry based on research and development. The development of new drugs requires a large number of scientific researchers and huge capital investment (36). If an enterprise has good development prospects, strong expansion ability, and rapid development speed, its profitability and financial situation are relatively excellent (37). Operation capability is the daily working capital used by enterprises for business turnover, which can be extended to all aspects of enterprise production and operation. Practice shows that only when the operation capability is well operated, the enterprise's investment, production, and sales can be in a virtuous cycle, maximize the enterprise value, and obtain higher profitability (38). Obviously, CM is also an important indicator of profitability (39). From the cash flow situation, we can see the profitability, debt repayment, financing, and other capabilities of the enterprise to a certain extent (40). Enterprises can attract more customers through marketing to improve their net profit (41). The ownership structure determines the shareholder structure, the degree of ownership concentration, and the identity of major shareholders, resulting in great differences in the ways and effects of shareholders exercising their power, which directly affects the corporate governance model and the profitability of enterprises (42). If the ratio of debt to equity is too high, it will bring too high financial risk to the enterprise; If it is too low, it cannot make full use of its debt ability and the hidden benefits of tax brought by debt, which will directly affect its profitability (43). Table 1 shows the descriptive statistics of the variable. The average incidence of profitability is 10.819, which deserves attention. The SDs for R&D capability (*RD*) and capital structure (ALR) show that they change greatly over time. The empirical analysis uses the natural logarithm of the variables.

**Table 1:** Descriptive statistics for variables.

**Variables**	**Mean**	**Min**	**Max**	**Std**.
Profitability (*Pro*)	10.819	11.170	10.289	0.293
R&D capability (*RD*)	3.860	3.410	4.380	0.180
Growth capability (*GRO*)	12.180	5.650	13.870	1.170
Operation capability (*ART*)	10.690	8.870	13.060	1.200
Cost management (*CM*)	2.140	1.020	4.180	0.670
Cash flow management (*CFM*)	18.230	14.100	21.360	0.350
Marketing capability (*SM*)	5.010	3.410	6.970	1.220
Ownership structure (*PSH*)	11.470	5.480	14.940	1.930
Capital structure (*ALR*)	12.180	5.650	13.870	1.740

## Empirical Results

### DID Empirical Results

Before the DID test, it is necessary to test the applicability of the treatment group and the control group. Only when the applicability test is passed, the estimation result can be judged to be accurate. An important premise of the applicability test is that the net profits of the treatment group and the control group have the same trend before the centralized drug procurement policy is proposed. Although there are differences between the treatment group and the control group, it can be judged that the control group is appropriate as long as their development trend is consistent before the centralized drug procurement policy is put forward and the difference in profitability between bid-winning enterprises and non-bid-winning enterprises is fixed. The regression model of parallel trend test is:

(12) $$\begin{array}{r} pro_{it}=\alpha_{0}+\overset{2017}{\underset{t=2011}{\sum }}\alpha_{t}DU\cdot DT_{t}+\gamma x_{it}+\eta_{t}+\mu_{i}+\varepsilon_{it} \end{array}$$

Based on this, the time dummy variable *DT* from 2011 to 2017 is assigned respectively. When t = 2017, *DT* = 1, and the rest *DT* = 0, indicating the time difference of the year before the implementation of drug centralized purchase policy of China. By analogy, when t = 2012, t = 2011,…, t = 2003, assign values to *DT*, respectively. The assignment rule of individual dummy variable *DU* remains unchanged. The value of listed companies participating in China's drug centralized purchase policy is 1, and the value of other listed companies not participating is 0. *DU* · *DT* is the product of *DU* and *DT*. The coefficient represents the intergroup difference between the experimental group and the control group before the implementation of drug centralized purchase policy of China (2018-t), that is, the parallel trend (44–47).

The results are shown in Table 2. α_*t*_ all are not significant, which indicates that the parallel trend between the control group and the experimental group was established before the proposal of China's drug centralized purchase policy. Before the policy is put forward, the changing trend of the net profit of the bid-winning enterprises and the non-bid-winning enterprises is basically the same, so it can be determined that the non-bid-winning enterprises are reasonable as the control group.

**Table 2:** Parallel trend test.

**Variables**	**Coefficient**	***t*-value**	**Conclusion**
α_2017_	0.020	0.650	Not significant
α_2016_	−0.043	−1.300	Not significant
α_2015_	−0.007	−0.220	Not significant
α_2014_	−0.010	−0.290	Not significant
α_2013_	−0.011	−0.330	Not significant
α_2012_	−0.006	−0.180	Not significant
α_2011_	−0.005	−0.160	Not significant

In order to further observe how the policy effect changes over time, this paper uses “progressive” did to explain the dynamic effect of China's centralized drug purchase policy on enterprise net profit. The progressive double difference model is as follows:

(13) $$\begin{array}{r} pro_{it}=\alpha_{0}+\alpha_{1}DID_{2018}+\alpha_{2}DID_{2019}+\alpha_{3}DID_{2020}\\ +\gamma x_{it}+\eta_{t}+\mu_{i}+\varepsilon_{it} \end{array}$$

Where the main concern coefficients are α_1_, α_2_, α_3_, if α_1_ is significantly negative, indicating that centralized drug procurement policy of China has significantly reduced the net profit of the enterprise in the year of implementation, and there is no time lag. if α_2_ and α_3_ are also significantly negative, indicating that drug centralized procurement policy of China has time sustainability and still has a negative impact on the net profit of enterprises in the years after the implementation of the policy.

The benchmark regression results are shown in Table 3. Time effect, individual effect, and combined effect are fixed in model 1. The coefficient of *DID* is −2.871, which is significant at the level of 1%. This shows that compared with non-bid-winning enterprises, the net profit of bid-winning enterprises has decreased significantly. Model 2 adds control variables to model 1, which not only significantly increases the goodness of fit of the model. The effect of centralized drug procurement policy of China on reducing the net profit of enterprises is still obvious, and the coefficient of *DID* is −1.351, which is still significant at the level of 1%. The regression results show that whether the control variable is added or not, China's centralized drug procurement is not conducive to the improvement of enterprise net profit. As shown in model 3, the coefficient of *DID*_2018_ is not significant, while the coefficients of *DID*_2019_ and *DID*_2020_ are significantly negative. The coefficient of the first year is not significant, which indicates that the negative impact of drug centralized procurement policy of China on the net profit of enterprises is not obvious in the year when enterprises win the bid. After the government officially purchases from pharmaceutical enterprises, the negative impact of drug centralized procurement policy of China on the net profit of enterprises begins to appear gradually (48–51). By observing the absolute number of its coefficient, it can be found that it is an increasing trend on the whole, indicating that the negative impact of China's drug centralized procurement policy on the net profit of enterprises is gradually increasing and has a lasting effect.

**Table 3:** Full sample regression results.

**Variables**	**Model(1)**	**Model(2)**	**Model(3)**
*DID*	−2.871^***^	−1.351^***^	-
DI*D*_2018_	-	-	−1.377
DI*D*_2019_	-	-	−3.685^***^
DI*D*_2020_	-	-	−8.160^***^
*C*	4.719^***^	6.453^***^	8.034^***^
Control variable	No	Yes	Yes
Individual fixation effect	Yes	Yes	Yes
Time fixed effect	Yes	Yes	Yes
*R^2^*	0.632	0.683	0.686

*** *Represent the significance levels of 1%*.

### Robustness Check

In this paper, the explanatory variable—enterprise net profit—is changed to net profit after deducting non-recurring gains and losses, and the regression is carried out by the DID method. In the test results, the *DID* coefficient is significantly negative at the level of 1%, indicating that the regression result in this paper is robust. Therefore, the robustness check takes net profit after deducting non-recurring gains and losses as the dependent variable in Table 4.

**Table 4:** Robustness check.

**Variables**	**Net profit after deducting non-recurring gains and losses**
*DID*	−2.356^***^
*C*	5.230^***^
Control variable	Yes
Individual fixation effect	Yes
Time fixed effect	Yes
*R^2^*	0.852

*** *Represent the significance levels of 1%*.

Based on the above robustness test, this paper further uses the counterfactual method to carry out the placebo test. By artificially setting a pilot time point of drug centralized procurement policy of China, the reduction of enterprise profits is tested. If the coefficient of *DID* is not significant, it indicates that the decline of enterprise profits is caused by centralized drug procurement policy of China, rather than other factors. On the contrary, the conclusion is not stable. The results in Table 5 show that by setting different pilot time points of China's drug centralized procurement policy, *DID* coefficients are no longer significant, indicating that the decline of enterprise profits is indeed caused by drug centralized procurement policy of China (52–55). So far, through the above robustness test, it is reasonable to believe that the estimation results and conclusions in this paper are very robust. The regression model of DID method after counterfactual transformation is set as follows:

(14) $$\begin{array}{r} pro_{it}=\beta_{0}+\beta_{1}DU\cdot DT+\gamma x_{it}+\eta_{t}+\mu_{i}+\varepsilon_{it} \end{array}$$

The implementation time of the policy is 2017 and 2016, respectively. The test results are far from the benchmark regression, which proves that the benchmark regression results are reliable.

**Table 5:** Counterfactual analysis.

**Variables**	**Coefficient**	***t*-value**	**Conclusion**
*DU* · *DT*_2017_	−2.156	−0.898	not significant
*DU* · *DT*_2016_	−1.378	−0.759	not significant
Control variable	Yes		
Individual fixation effect	Yes		
Time fixed effect	Yes		
*R^2^*	0.733		

## Conclusions

This paper comprehensively considers the heterogeneous purchase preference of medical institutions and the bargaining power of pharmaceutical enterprises. The effects of enterprise bargaining power and centralized procurement efficiency on the willingness of pharmaceutical enterprises to participate in centralized procurement were studied. Through the comparative analysis of four possible bargaining equilibrium results, this paper discusses the impact of centralized procurement regulation on drug procurement price, the procurement cost of medical institutions, and profits of pharmaceutical enterprises.

Based on the panel data of 65 generic drug-listed companies in China from 2011 to 2020, this paper uses the double-difference method to evaluate the impact of drug centralized procurement policy of China on the net profit of enterprises and draws the following conclusions: first, before the implementation of the policy, the difference between successful enterprises and unsuccessful enterprises is not obvious. After the implementation of the policy, the net profit of the winning enterprise decreased significantly (25, 56–58). Therefore, centralized drug procurement policy of China is not conducive to the improvement of enterprise net profit in the short term, and the effect is more obvious with the increase of policy implementation years.

In the face of the substantial increase in product supply, can the bid-winning enterprises keep up with the production capacity and quality? How to control the cost? Can you win the bid next year? Are worth thinking about. For generic drug manufacturers, the earlier the layout consistency evaluation is, the more favorable it will be. In the future, the consistency evaluation may face more fierce competition and more rigorous price negotiation. If it fails to pass the consistency evaluation, if the pilot is expanded to the whole country in the future, it may face no products to participate in the core market competition, and for the remaining 30–40% of the market, it is even more difficult to add the original research and exclusive varieties. Small enterprises without high-quality products, production capacity, and core competition will be eliminated one after another.

## Data Availability Statement

The original contributions presented in the study are included in the article/supplementary material, further inquiries can be directed to the corresponding author.

## Author Contributions

All authors listed have made a substantial, direct, and intellectual contribution to the work and approved it for publication.

## Conflict of Interest

H-QZ was employed by company ABB China. The remaining authors declare that the research was conducted in the absence of any commercial or financial relationships that could be construed as a potential conflict of interest.

## Publisher's Note

All claims expressed in this article are solely those of the authors and do not necessarily represent those of their affiliated organizations, or those of the publisher, the editors and the reviewers. Any product that may be evaluated in this article, or claim that may be made by its manufacturer, is not guaranteed or endorsed by the publisher.
